# Pleural Mesothelioma: Advances in Blood and Pleural Biomarkers

**DOI:** 10.3390/jcm12227006

**Published:** 2023-11-09

**Authors:** Claudio Sorino, Michele Mondoni, Giampietro Marchetti, Sergio Agati, Riccardo Inchingolo, Federico Mei, Sara Flamini, Filippo Lococo, David Feller-Kopman

**Affiliations:** 1 Division of Pulmonology, Sant’Anna Hospital of Como, University of Insubria, 21100 Varese, Italy; claudio.sorino@asst-lariana.it (C.S.); sergio.agati@asst-lariana.it (S.A.); 2 Respiratory Unit, ASST Santi Paolo e Carlo, Department of Health Sciences, Università degli Studi di Milano, 20122 Milan, Italy; 3 Pulmonology Unit, ASST Spedali Civili, 25123 Brescia, Italy; marchetti.giampietro@libero.it; 4 Pulmonary Medicine Unit, Department of Medical and Surgical Sciences, Fondazione Policlinico Universitario Agostino Gemelli IRCCS, 00168 Rome, Italy; riccardo.inchingolo@policlinicogemelli.it; 5 Respiratory Diseases Unit, Department of Internal Medicine, Azienda Ospedaliero Universitaria delle Marche, 60126 Ancona, Italy; federico.mei@ospedaliriuniti.marche.it; 6 Departement of Thoracic Surgery, Università Cattolica del Sacro Cuore, 00168 Rome, Italy; sara.flamini@guest.policlinicogemelli.it (S.F.); filippo.lococo@policlinicogemelli.it (F.L.); 7 Thoracic Surgery, Fondazione Policlinico Universitario Agostino Gemelli IRCCS, 00168 Rome, Italy; 8 Department of Medicine, Geisel School of Medicine at Dartmouth, Hanover, NH 03755, USA; david.j.feller-kopman@hitchcock.org; 9 Division of Pulmonary and Critical Care Medicine, Dartmouth-Hitchcock Medical Center, Lebanon, NH 03766, USA

**Keywords:** mesothelioma, pleural effusion, biomarkers, mesothelin, fibulin-3

## Abstract

Pleural mesothelioma (PM) is a type of cancer that is highly related to exposure to asbestos fibers. It shows aggressive behavior, and the current therapeutic approaches are usually insufficient to change the poor prognosis. Moreover, apart from staging and histological classification, there are no validated predictors of its response to treatment or its long-term outcomes. Numerous studies have investigated minimally invasive biomarkers in pleural fluid or blood to aid in earlier diagnosis and prognostic assessment of PM. The most studied marker in pleural effusion is mesothelin, which exhibits good specificity but low sensitivity, especially for non-epithelioid PM. Other biomarkers found in pleural fluid include fibulin-3, hyaluronan, microRNAs, and CYFRA-21.1, which have lower diagnostic capabilities but provide prognostic information and have potential roles as therapeutic targets. Serum is the most investigated matrix for biomarkers of PM. Several serum biomarkers in PM have been studied, with mesothelin, osteopontin, and fibulin-3 being the most often tested. A soluble mesothelin-related peptide (SMRP) is the only FDA-approved biomarker in patients with suspected mesothelioma. With different serum and pleural fluid cut-offs, it provides useful information on the diagnosis, prognosis, follow-up, and response to therapy in epithelioid PM. Panels combining different markers and proteomics technologies show promise in terms of improving clinical performance in the diagnosis and monitoring of mesothelioma patients. However, there is still no evidence that early detection can improve the treatment outcomes of PM patients.

## 1. Introduction

Pleural mesothelioma (PM) is a rare type of cancer that originates from the pleural mesothelial cells, although mesothelioma can also be seen in the peritoneum, pericardium, and tunica vaginalis. Most cases (over 80%) occur in males and are associated with asbestos exposure. Asbestos is thought to cause prolonged pleural inflammation, free radical production, interference with mitosis, and activation of proto-oncogenes. Due to a latency period of several decades between inhalation of the fibers and the onset of symptoms, many cases are detected when patients have advanced-stage disease and their clinical condition is poor [1].

According to the 2021 WHO Classification of Tumors of the Pleura, the prefix “malignant” has been omitted from localized and diffuse pleural mesothelioma because all mesotheliomas are regarded as malignant, while the well-differentiated papillary mesothelioma (WDPM) has been renamed as a well-differentiated papillary mesothelial tumor (WDPMT) given its relatively indolent behavior [2].

Besides asbestos inhalation, associations with ionizing radiation, such as mantle radiation for Hodgkin’s lymphoma or a germline mutation of the BRCA 1-associated protein (BAP1), are known risk factors [3]. An increase in the incidence of PM cases without a concomitant increase in asbestos exposure suggests that genetic predisposing factors may play a crucial role, and idiopathic/spontaneous cases are thought to account for a significant percentage of cases [4,5,6].

The most frequent imaging features of PM are pleural thickening and pleural effusion. Furthermore, PM often shows a high uptake to 18F-FDG PET (sensitivity of 88–95%), which also has a relevant prognostic value [7].

Histology can distinguish three main subtypes of PM: epithelioid (the most frequent), sarcomatoid (the most aggressive), and biphasic. Calretinin, Wilms’ tumor gene (WT1), cytokeratin 5/6 (CK5/6), and D2-40 are the most useful mesothelial immunohistochemical markers to support an PM diagnosis [8].

The current treatment options are very limited, leaving PM as an incurable disease with an overall poor prognosis. Recent reports indicate a slightly improving trend in survival 1 year and 3 years after diagnosis (from 38% to 40% and from 7% to 10%, respectively), despite an increase in median age at the time of diagnosis (from 75 to 76 years old) [9].

The treatment of mesothelioma should be discussed by a multidisciplinary team, taking into consideration histology, staging, age, comorbidities, performance status, and patient preferences [10].

Radical surgery for PM has been proposed in multimodal approaches, which combine it with other treatments such as chemotherapy, radiotherapy, and immunotherapy. The main surgical treatments include extrapleural pneumonectomy (EPP), which is the en bloc resection of the pleura and lung, and pleurectomy/decortication, in which the lung is kept in situ. The former is used less and less often due to its high morbidity. However, to date, no phase 3 study has comprehensively shown an advantage of radical surgery. Consequently, according to the main international guidelines, multimodal treatment should only be performed in dedicated high-volume mesothelioma centers with specific surgical and multidisciplinary expertise [11].

For many years, the management of PM was limited to best supportive care (BSC) or platinum-based single-agent chemotherapy, until the trial by Vogelzang et al. in 2003 demonstrated an improvement in overall survival and quality of life by combining cisplatin and pemetrexed, a folate antimetabolite [12]. More recently, further improvements in survival were found when bevacizumab, an anti-VEGF inhibitor, was added to therapy regimens [13]. Finally, immunotherapy has emerged as a possible therapeutic strategy for PM. Following the results of the phase 3 trial CheckMate, the combination of nivolumab and ipilimumab is considered the most effective treatment today, and has been approved by the Food and Drug Administration (FDA) as the first-line regimen for patients with advanced-stage PM [14,15].

The search for biomarkers of PM has long raised much interest, with three main potential purposes: (1) screening in people at risk (both exposed to asbestos and family members in “genetic-related” PM cases); (2) improving the process of diagnosis in patients with pleural effusion or other abnormalities, such as pleural thickening; and (3) assessing responses to treatment and prognostic evaluation. In this context, the detection of soluble or pleural fluid biomarkers of PM could be useful in reducing the need for invasive procedures in patients with poor performance status. The 2018 British Thoracic Society Guidelines for the investigation and management of PM recommend considering biomarker testing only in patients with suspicious cytology who are not fit enough for more invasive diagnostic tests, and do not suggest them in isolation to screen or diagnose PM, nor to predict treatment response or survival [16]. The 2020 European Respiratory Society (ERS/European Society of Thoracic Surgeons (ESTS)/European Association for Cardio-Thoracic Surgery (EACTS)/European Society for Radiotherapy and Oncology (ESTRO)) for the management of PM does not suggest the routine determination of mesothelin or other biomarkers for diagnosis, screening, or prognostic assessment in the absence of clear scientific evidence regarding their utility [17].

Screening methods should be minimally invasive and cost-effective tests that are able to identify PM among asbestos-exposed populations in order to potentially treat the disease at earlier stages. Although it is desirable to have very sensitive and specific tests, for the screening of asymptomatic subjects, a high specificity is strongly recommended to reduce the number of false positives, as well as the consequent unnecessary procedures and psychological stress for the subjects involved [18].

A high specificity is also necessary for tests concerning the differential diagnosis of pleural lesions, the greatest of which is obtained with pleural biopsies. The complication rate, morbidity, and costs associated with pleural biopsies depend on the procedure utilized to attain the specimen, such as medical thoracoscopy, thoracic surgery, and transthoracic imaging-guided biopsy [19].

Despite being relatively invasive, medical or surgical thoracoscopy may allow for diagnosis and staging and, at the same time, offer definitive treatments to counteract the recurrence of malignant pleural effusion (i.e., placement of an indwelling pleural catheter and/or palliative pleurodesis) [20]. The diagnostic definition of PM and its distinction from non-malignant lesions are crucial for making management decisions involving the patient and family members.

In the last two decades, several studies have investigated diagnostic and prognostic markers for PM, yet to date, the most reliable predictors of disease outcome are still the clinical and pathological parameters. In particular, non-epithelioid histology, poor performance status, male gender, anemia, thrombocytosis, leukocytosis, elevated LDH, older age, and advanced disease are poor prognostic indicators in patients with PM. Many of these parameters are included in the more widely used score systems for PM, namely, that of the European Organization for Research and Treatment of Cancer (EORTC) and Cancer and Leukemia Group B (CALGB). They have been introduced over 20 years ago and remain valid, although many studies have attempted to update them, identify additional prognostic biomarkers, and develop models that combine clinical and molecular features [21,22].

A further, and very interesting, potential implication of mesothelioma biomarkers is the identification of therapy targets.

Candidate biomarkers can be molecules with different characteristics, such as proteins or their fragments, nucleic acids, lipids, and metabolites. As a result, methods for their identification vary greatly. Most protein studies have used enzyme-linked immunosorbent assays (ELISAs).

Proteomics-based approaches, such as those based on mass spectrometry, are promising tools and have been increasingly implemented to identify and quantify biomolecules in a variety of biological samples. They require three essential steps: protein extraction and separation, protein identification, and protein verification.

The evaluation of nucleic acids is more complex, as it requires the extraction, quantification, and purification of RNA, with the samples stored at very low temperatures (−80 °C). The purified RNA is then reverse-transcribed into cDNA, which, in turn, is amplified by a polymerase chain reaction (PCR).

The evaluation of nucleic acids and, even more so, proteomics has high costs and poses accessibility problems in low-income countries.

Table 1 summarizes some of the common methods used for identifying biomarkers, along with their pros and cons [23]. The choice of method depends on research goals, available resources, and the characteristics of the biomarkers in question. Combining multiple techniques often provides a more comprehensive understanding and increases the chances of discovering robust biomarkers. Validation through experiments and clinical studies is essential to ensure the reliability and relevance of the identified biomarkers.

## 2. Methods

We carried out a non-systematic and comprehensive narrative literature review. The search engines PubMed and Cochrane Library were used to retrieve the most relevant articles on the aforementioned topic from its origin to 26 August 2023. The following keywords were combined to address our research question: pleural mesothelioma, biomarker, asbestos-related disease, pleural fluid, accuracy, screening, diagnosis, and monitoring. We then thoroughly analyzed the bibliographies of relevant studies to identify additional potentially eligible studies.

The inclusion criteria were original articles in the English language, as well as clinical trials (randomized, prospective, or retrospective). The abstracts identified by this search were independently reviewed by two authors (C.S. and F.L.), and discrepancies were resolved by a third author (M.M.). The selected articles were examined in full, then processed and summarized.

Furthermore, we searched “ClinicalTrials.gov” (accessed on 30 October 2023) for ongoing studies using the keywords “Pleural Mesothelioma AND Biomarkers” and their synonyms, then analyzed and manually selected the relevant trials.

## 3. Pleural Fluid Biomarkers of PM

Multiple molecules found in pleural fluid have been investigated as potential biomarkers of malignancy. This would be a less invasive and relatively less expensive technique than pleural biopsies [24].

In particular, pleural fluid biomarkers are potentially promising diagnostic tools for PM, which, in about half of cases, occurs with pleural effusion. However, the diagnostic value of the most studied biomarkers for PM in pleural effusion is still largely to be defined [17].

In this context, the greater attention of the scientific community has been directed toward mesothelin and soluble mesothelin-related peptides (SMRPs), fibulin-3, osteopontin, and cell-free microRNA (Table 2).

In a recent meta-analysis on the markers of PM, 36% of the included studies researched them in pleural effusions. Proteins were the most investigated biomarkers (89%), while DNA and miRNA were studied in only 5.5% [25].

### 3.1. Mesothelin

Mesothelin is a protein that is normally present on the mesothelial cells of the pleura, peritoneum, and pericardium. It appears to play a role in cell adhesion, but is likely a nonessential component in normal cells. Mesothelin is overexpressed in some malignancies, such as PM, but also in pancreatic, ovarian, and lung adenocarcinomas [47].

This fact has made it an attractive candidate as a biomarker for the diagnosis of PM, for screening people exposed to asbestos, for monitoring the progression of the disease, and as a potential target for cancer therapy [48].

The full-length human mesothelin gene (Full-ERC/mesothelin) encodes a 71-kDa precursor protein, which is cleaved into two products, a 40-kDa C-terminal fragment (C-ERC/mesothelin) and a 31-kDa N-terminal fragment (N-ERC/mesothelin, also known as megakaryocyte potentiating factor, MPF). Both mesothelin and related peptides (SMRPs), including MPF, have been found in human serum and pleural effusion. As a result, assays have been developed to determine their pleural and blood levels (see also the next section on “Serum biomarkers of PM”) [49].

Mesothelin was originally known as the CAK1 antigen. It was identified by the murine monoclonal antibody K1, and can now be identified by “second generation” anti-mesothelin antibodies such as the 5B2 clone.

A pioneering study of mesothelin using K1 found that, among 23 patients with PM, all 15 individuals with the epithelioid subtype had mesothelin expression; the 4 with the sarcomatous subtype were all negative; and in the 4 patients with biphasic PM, only the epithelial component stained for mesothelin. In the same study, none of 23 lung adenocarcinomas with different degrees of histologic differentiation demonstrated reactivity with the K1 antibody [50].

Similar results were subsequently found using the antimesothelin antibody 5B2 in paraffin-embedded PM tissue samples [51]. Out of the 55 mesothelioma specimens which were studied, mesothelin reactivity was observed in all 44 epithelioid PM and in the 3 epithelial components of biphasic PM. Conversely, none of the 8 sarcomatous mesotheliomas expressed mesothelin. Despite the limited case studies, the results provide further evidence that mesothelin is present in all epithelial mesotheliomas and is absent in the sarcomatous type. Consequently, a positive mesothelin immunostain suggests an epithelioid PM, although not absolutely specific, whereas a negative mesothelin immunostain strongly argues against the diagnosis of epithelioid PM [52].

The meta-analyses by Gao et al. [26] and Cui et al. [27] evaluated the diagnostic accuracy of SRMPs on pleural fluid for the diagnosis of PM. They analyzed the SMRP concentrations in 13 and 11 studies, respectively, and all of them reported higher levels of mesothelin in PM patients compared to controls. As meta-analyses, the cutoff values in the included articles were quite different, ranging from a minimum of 8 nmol/L to a maximum of 24.1 nmol/L. Therefore, Gao et al. divided the studies into two groups, with 15 nmol/L as the boundary, but this did not significantly reduce the heterogeneity. In the two meta-analyses, the summary estimates of sensitivity were 0.68 and 0.79, the specificity values were 0.91 and 0.85, the positive likelihood ratios were 7.8 and 4.78, the negative likelihood ratios were 0.35 and 0.30, and the diagnostic odds ratios were 22 and 19.50, respectively. As expected, the specificity with a cut-off value of 20 ± 0.4 nmol/L was higher than with a cut-off value of 12 ± 0.6 nmol/L and 8 ± 0.6 nmol/L, and had a range of 83.7% to 97.1%. Altogether, the authors concluded that PM can be suspected when the SMRPs in pleural effusion are higher than 8 nmol/L, whereas values higher than 20 nmol/L, are strongly suggestive.

A more recent meta-analysis by Schillebeeckx et al. [25] included 19 studies evaluating the diagnostic effectiveness of pleural effusion mesothelin. The cut-off values which were ranged from 6 nM (where sensitivity and specificity were, respectively, 72% and 46%) to 50 nM (with sensitivity and specificity of 44% and 100%). The most commonly used cut-off values fell around 20 nM, with sensitivities ranging from 44% to 100% and specificities from 46% to 100%, while the overall AUC_ROC_ was 0.83 (95% CI: 0.81–0.85).

### 3.2. Fibulin-3

Fibulin-3 is a glycoprotein encoded by the epidermal growth factor-containing fibulin-like extracellular matrix protein 1 gene. It plays a role in cell proliferation and migration [53,54].

Fibulin-3 has low expression in normal tissues, but it is overexpressed in several cancers, including PM, and is also secreted in body fluids. It accumulates in the pleural effusions of PM patients and has been proposed to distinguish these patients from individuals who have non-malignant pleural inflammation. An AUC_ROC_ of 0.93 was found when pleural fibulin-3 values were evaluated to discriminate mesothelioma from both benign and malignant effusions, with cutoffs for maximum sensitivity and specificity between 378 ng per milliliter and 346 ng per milliliter [28]. These results were not confirmed by other studies that found similar fibulin values in effusions from mesothelioma and other diseases [55]. The overall AUC_ROC_ found in the meta-analysis by Schillebeeckx et al. was 0.68 (95% CI: 0.50–0.87) [25].

A comparative analysis has suggested that fibulin-3 correlates less accurately than mesothelin with PM diagnosis, whether measured in plasma or pleural effusion; thus, mesothelin has been recognized as the best pleural marker that is usable for routine diagnostic purposes [56,57].

Conversely, fibulin-3 has been proposed as a better prognostic factor of PM, since recent evidence suggests that fibulin-3 promotes the malignant behavior of mesothelial cells, whereas fibulin-3 knockdown decreases viability, clonogenic capacity, and invasion, as well as chemoresistance, in PM cells.

### 3.3. Hyaluronic Acid

Hyaluronan, or hyaluronic acid (HA), is a large polysaccharide that contributes to the progression of several types of cancer [58]. It has been shown to be elevated in mesothelioma-associated pleural effusions [29], although several studies have suggested that its increase is mostly due to the release of growth factors from tumor cells that may stimulate other cells to produce HA [59]. HA is rapidly removed from circulation by the clearance receptor stabilin-2, and has a plasma half-life of 2.5–5 min.

A cutoff of 100,000 ng/mL showed a sensitivity of 44.0% and a specificity of 96.5% for differentiating effusions due to mesothelioma from those induced by other causes, with an AUC_ROC_ curve of 0.832 [29].

In the past, high technical expertise was required to measure hyaluronic acid by high-performance liquid chromatography, and this has limited the number of studies on this biomarker. More recent studies using more intuitive test systems have demonstrated that the mesothelin and hyaluronic acid levels in pleural effusion have similar levels of diagnostic accuracy, and that combining the two markers in a two-step model improves diagnostic accuracy [60].

### 3.4. Cell-Free microRNAs

MicroRNAs are short, noncoding, single-stranded RNA molecules that regulate gene expression at the post-transcriptional level.

MicroRNAs affect the courses of many important processes of the human organism, including cell division, proliferation, differentiation, apoptosis, and the formation of blood vessels. Altered expression of microRNAs has been shown in several cancers, which suggests a potential oncogenic or suppressor role [61].

Some studies have evaluated the serum levels of different microRNAs as markers of malignant mesothelioma, while only the study by Birnie et al. has analyzed them in pleural fluid [30]. The authors analyzed microRNAs in the pleural effusion cells and supernatants from 26 patients with PM and 21 with pleural effusion due to non-PM conditions. They found that four microRNAs (miR-944, miR-139-5p, miR-210, and miR-320) found in pleural effusion were upregulated, and seven (miR-200b, miR-200c, miR-143, miR-200a, miR-203, miR-31, and miR-874) were downregulated. A combination of miR-143, miR-210, and miR-200c was able to differentiate PM from non-PM with an AUC_ROC_ of 0.92.

### 3.5. CYFRA-21-1 and CEA

CYFRA-21-1 is the soluble fragment of cytokeratin 19. It can be released into circulation after cell death, thus exhibiting a close relationship with tumor cell necrosis and apoptosis. CYFRA-21-1 is found in the blood of patients with different epithelial malignancies, including non-small-cell lung cancer (NSCLC), and has been used to predict diagnosis and prognosis [62]. Although CYFRA-21-1 has not been extensively investigated in PM, all studies measuring it in pleural effusion found higher levels in PM patients compared to controls [31,63,64]. However, the diagnostic accuracy was modest, with AUC_ROC_ values ranging from 0.65 to 0.76.

CEA is a glycoprotein involved in cell adhesion. In healthy individuals, very low levels of CEA are detectable in the bloodstream and body fluids, while its increase has been reported in several cancers and non-cancerous conditions. Two studies measuring CEA in pleural fluid reported its increase in PM [31,65], while another study demonstrated that pleural CEA in PM was less elevated than in other cancer types, suggesting that CEA levels above 3 ng/mL in pleural fluid may exclude the diagnosis of PM [64]. The overall AUC_ROC_ is 0.55; therefore, CEA is currently of poor diagnostic accuracy and is not recommended as a differential diagnostic biomarker for PM [25].

### 3.6. Combined Markers Panels

In a study aiming to establish a predictive model using biomarkers from pleural effusions, samples from 190 consecutive patients were collected [66]. The biomarkers significantly associated with PM were hyaluronan, N-ERC/mesothelin, C-ERC/mesothelin, and syndecan-1. A two-step model using hyaluronan and N-ERC/mesothelin yielded good discrimination, with an AUC_ROC_ of 0.99 (95%CI: 0.97–1.00) in the model generation dataset and 0.83 (0.74–0.91) in the validation dataset, respectively.

Recently, a novel affinity-enrichment mass spectrometry-based proteomics method was applied for the explorative analysis of pleural effusions from a prospective cohort of 84 individuals who underwent thoracoscopy due to suspected PM [67]. The immunohistology of the pleural biopsies confirmed PM in 40 patients and ruled out PM in 44. The authors identified protein biomarkers with a high capability to discriminate PM from non-PM patients and applied a random forest algorithm for the purpose of building classification models. Depending on the specific protein combination, the proteomic analysis of pleural effusions identified panels of proteins with excellent diagnostic properties (90–100% sensitivities, 89–98% specificities, and AUC_ROC_ 0.97–0.99). Proteins associated with cancer diagnosis included galactin-3 binding protein, testican-2, haptoglobin, Beta ig-h3, and protein AMBP. Furthermore, the study confirmed the previously reported diagnostic accuracies of the PM markers fibulin-3 and mesothelin. Subsequent studies should validate these findings in separate cohorts of patients and investigate the possible impact of PM subtypes on biomarker selection, as well as the implementation of machine learning in the mass-spectrometry-based diagnosis of PM.

### 3.7. Cytology

The detection of neoplastic invasion has always been a key element in diagnosing PM with certainty, but diagnosis based solely on pleural effusion cytology is controversial, mainly due to poor sensitivity. When a large amount of pleural fluid is submitted for cytological evaluation, the pathologist can prepare cell-block sections for immunohistochemical investigation and obtain a high level of specificity [68].

The best interpretative yield derives from the correlation of the cytological results with the imaging, which can provide information on the anatomical distribution of the lesion, evidence of the nodularity of the pleural disease, and, sometimes, tissue invasion.

Although “positive” and “negative” immunohistochemical markers have been shown to be remarkably effective in distinguishing between epithelioid mesothelioma and other secondary malignancies, no biomarker has 100% sensitivity or specificity for diagnosing mesothelioma.

The 2021 WHO classification of tumors of the pleura recommends specifying the specimen type in diffuse PM reports (e.g., extended pleurectomy/decortication; extrapleural pneumonectomy; and other smaller specimens, including small biopsy specimens and cytology). Regarding histology, PM can be determined directly by morphology through hematoxylin–eosin staining. Nonetheless, pathologists usually recommend confirmation through immunohistochemistry. Calretinin, Wilms tumor 1 (WT-1), cytokeratin 5 (CK5), podoplanin, mesothelin, and heart development protein with EGF-like domains 1 (HEG1) are immunohistochemical biomarkers of mesothelial differentiation, whereas carcinoembryonic antigen (CEA), B72.3, Ber-EP4, Lewisy blood group (BG8), MOC-31, CD15, mucin-4 (MUC4), and claudin-4 are markers suggestive of epithelial metastasis [2].

The loss of BAP1 protein expression by immunohistochemistry has recently been suggested as a potential marker for identifying MM, as it has been observed in more than half of PM, either epithelioid, biphasic, or sarcomatoid [69].

Recent advances in cytological analysis promise diagnostic advances for PM. Biancosino et al. The study cited in [70] analyzed 5731 specimens of pleural effusions from 4552 patients, of which 444 were diagnosed as PM. Cytological evaluation achieved a sensitivity of 0.50 and specificity of 0.99 for PM diagnosis. The supplemental assessments of HA (above 30 mg/L) raised the sensitivity to 0.70 without affecting the specificity. The authors concluded that the cytological evaluation of pleural effusions aided by the assessment of HA has a diagnostic accuracy for PM that is no less than that of the standard histological evaluation, and may be considered in difficult or doubtful diagnostic cases.

Similarly, a large monocentric database was retrospectively explored in order to clarify the value of cytology in distinguishing malignant mesothelioma according to the International System for Reporting Serous Fluid Cytopathology (ISRSFC) [71]. Cytological samples were available for analysis in 210 patients with malignant mesothelioma (164 pleural and 46 peritoneal effusions). All cases were reviewed and reclassified according to the proposed ISRSFC scheme. The final histological diagnosis consisted of epithelioid mesothelioma in 192 (91.4%) patients, and sarcomatoid type in the remaining 18 (8.6%). The cytological cases were reclassified as follows: 2 (0.9%) as non-diagnostic, 81 (38.6%) as negative for malignancy, 4 (1.9%) as atypia of undetermined significance, 11 (5.2%) as suspicious for malignancy, and 112 (53.4%) as malignant. Sarcomatoid cells in the malignant category appeared solitary, with moderate or marked nuclear pleomorphisms and irregular chromatin if compared with the epithelioid subtype. The authors concluded that morphological features, coupled with clinical–radiological data, may help clinicians to adequately manage the patients.

## 4. Blood Biomarkers of PM

Serum is the most investigated matrix for biomarkers of PM. Several serum biomarkers in PM have been studied: most commonly, mesothelin, osteopontin, and fibulin-3 [25,72] (Table 2).

### 4.1. Mesothelin and SMRP

Mesothelin has been the most studied serum biomarker in PM [73], with enzyme-linked immunosorbent assay (ELISA) being the most often employed analytical technique.

An individual meta-analysis of patient data on the diagnostic value of soluble mesothelin in 4491 patients estimated the sensitivity and specificity to be 47% and 95%, respectively, with an AUC_ROC_ of 0.77 [74].

A more recent systematic review and meta-analysis, which included 27 studies, showed an overexpression of the protein in patients with malignant mesothelioma, with cut-off thresholds ranging between 0.55 nM and 2.4 nM. Higher protein concentrations were detected in the epithelioid type than in other subtypes of PM [17]. AUC_ROC_ values were different according to the different control groups, with the highest (0.93–0.94) in controls including only benign pleural effusions, and the lowest when comparing serum mesothelin with other malignancies. This suggests a low specificity of the biomarker. AUC_ROC_ values of 0.74 and 0.81 were found in the case of stage I mesothelioma and all-stage disease, respectively, when compared with asbestos-exposed healthy controls. Notably, plasma mesothelin showed an overall AUC_ROC_ value of 0.86 [25].

Elevation of serum MPF in PM patients has also been reported, with a good correlation with mesothelin concentration and higher values in the epithelioid subtype [17,75]. However, a lower accuracy of MPF compared to mesothelin was reported (AUC_ROC_ of 0.78 and 0.81, respectively) [25,34,75].

Isolated MPF serum levels were found to be a predictor of poor survival in PM by Yu et al. [35].

Conflicting findings are present in the literature regarding the association of SRMP levels with prognosis. A meta-analysis of 8 studies based on 579 PM patients showed a good correlation between high SMRP levels and worse survival [76]. However, subsequent studies did not confirm these findings [77,78,79]. The use of different cut-off values across studies might partially explain this discrepancy [73].

Increased SMRP levels are related to large tumor volume [80,81] Several studies have demonstrated that longitudinal measurement may be used to assess tumor response and progression, and may be associated with radiological findings [82,83,84,85,86]. However, Katz et al. recently confirmed the correlation of SMRP and fibulin-3 with initial tumor volume, but recent studies have failed to demonstrate the clinical utility of the biomarkers in terms of assessing the tumor response in patients receiving immunotherapy [81].

SMRP levels decrease after surgery, and are a promising serum biomarker for the detection of recurrence after the resection of epithelial PM [73,87].

Mesothelin expression represents a key criterion for selecting patients to undergo mesothelin-targeted treatments. Indeed, it has also been studied as a potential therapeutic target in patients with PM. [73] Immunotherapeutic strategies targeting mesothelin, as well as the related study phases, are shown in Table 3.

### 4.2. Osteopontin (OPN)

Serum osteopontin shows good accuracy in terms of diagnosing PM when compared with healthy controls with or without asbestos exposure (AUC_ROC_ of 0.89 and 0.86, respectively), but has no utility when compared with benign pleural effusions [25,36] and other diseases [37], thus suggesting that this biomarker has low specificity.

Plasma OPN shows higher accuracy than serum markers, as suggested by meta-analyses and head-to-head studies [25,94,95,96]. This might be caused by the easy degradation by thrombin in peripheral blood [97].

OPN may play a prognostic role. Several studies have shown that high OPN levels are related to poor prognoses in patients with PM [38,39,40,95].

### 4.3. Fibulin-3

The performance characteristics of plasma fibulin-3 were first reported by Pass et al. in 2012 [28]. In 507 patients from 3 cohorts, the authors found a sensitivity and specificity of the biomarker of 95% and an AUC_ROC_ of 0.99 when distinguishing PM from healthy asbestos-exposed controls and patients with other malignancies. These findings were not confirmed by subsequent studies [57,98,99,100], which showed a lower accuracy of the biomarker.

However, a recent meta-analysis that included studies on plasma fibulin-3 showed an overall AUC_ROC_ of 0.91, which was the highest for plasma [25]. Head-to-head studies which compared the diagnostic performances of Fibulin-3 and mesothelin/SMRP showed conflicting and inconclusive findings [36,57].

Several studies have shown that serum/plasma fibulin-3 is not a reliable marker for prognosis [38] or for assessing the response to immunotherapy in PM [81].

Fibulin-3 has also been proposed as a relevant molecular target to reduce PM progression, and anti-fibulin-3 approaches are being studied [88].

### 4.4. Calretinin

Calretinin is a calcium-binding protein, which was originally found in neurons but is also expressed on the surfaces of mesothelial cells. Calretinin is widely used in immunohistochemical evaluations of cyto-histological specimens of suspected PM, both epithelioid and sarcomatoid [32].

Its detection in plasma and serum does not differ significantly, and higher circulating calretinin values have been detected in subjects with PM compared to healthy controls exposed to asbestos [101]. Studies on mouse primary mesothelial cells have suggested that the overexpression of calretinin would favor the proliferation and migration of mesothelial cells [102].

As a consequence, researchers have begun to hypothesize that calretinin may be a possible blood biomarker for screening, as well as a new potential therapeutic target of PM. Studies have shown promising findings regarding this marker for the early diagnosis of PM and in terms of distinguishing patients with PM from asbestos-exposed and healthy controls [33,103].

Furthermore, calretinin, both alone and in combination with mesothelin, was also evaluated in a large prospective cohort study on subjects with benign asbestos-related diseases who participated in annual screens. The combination of the two markers obtained a sensitivity and specificity of 46% and 98%, respectively, in the detection of mesothelioma up to about a year before clinical diagnosis [104].

### 4.5. MicroRNAs (miRNAs) and Long Non-Coding RNAs (lncRNAs)

MiRNAs are non-coding RNAs, 20–25 nucleotides in length, which regulate gene expression at the post-transcriptional level by binding to the 3’-untranslated regions of their mRNA targets and subsequently inhibit translation or induce cleavage. MiRNA expression signatures are associated with the tumor type and clinical outcome, as demonstrated by genome-wide profiling. Therefore, miRNAs have a potential role as candidates for diagnostic and prognostic biomarkers and as tools for therapeutic targets [105].

Circulating miRNA profiles of PM patients have been studied to identify markers for early detection, differential diagnosis, and prognosis.

Several studies have reported increases in the expression of miR-197-3p, miR-1281, miR-548-3p, miR-20a, miR-625-3p, and miR-34b/c alongside miR-126 downregulation, which can be attributed to its tumor-suppressive activity [17,90,106]. The findings from these studies indicate that various miRNAs have different AUC_ROC_ values. For instance, miR-197-3p had an AUC_ROC_ value of 0.76, miR-625-3p had an AUC_ROC_ value of 0.80, and miR-20a scored as high as 0.98 based on numerous studies [33,104]. Conversely, miR-126I represents one of the most frequently screened circulating miRNAs, possessing a highly precise serum marker with an AUC_ROC_ of 0.80. However, the best accuracy is found when compared with healthy controls without asbestos exposure [25]. When compared with healthy individuals with and without asbestos exposure, it shows a poor diagnostic value, with pooled sensitivity and specificity for PM of 71% and 69%, respectively (AUC_ROC_ 0.74) [91].

Weber et al. were able to demonstrate different levels of miR-132 expression in circulating samples from mesothelioma patients and asbestos-exposed control subjects. The discrimination sensitivity was 86%, and the specificity was 61%. When miR-132 was combined with the previously described miR-126, the sensitivity was 77% and the specificity was 86% [41].

To identify a novel miRNA signature in exosomes extracted from plasma, Cavalieri et al. used an OpenArray approach. In particular, the combination of miR-103a-3p and miR-30e-3p was able to discriminate PM from WEA (with a sensitivity of 95.8% and a specificity of 80%) [43].

The expression of different long non-coding RNAs (lncRNAs) in mesothelioma has been studied, and these have been proposed as potential biomarkers, such as ATG5 [44], GAS5 [45]. DRAM1 miRNA, ARSA miRNA, hsa-miR-2053, and lncRNA-RP1-86D1.3 [46]. The results of the studies are somewhat heterogeneous, and this can mainly be attributed to the use of diverse control groups, small sample sizes, and the lack of standardization in the detection methods of circulating microRNAs [24,73].

Because of their wide regulatory capabilities, microRNAs are potentially effective therapeutic targets (Table 3) [42,92,93,107].

### 4.6. Circulating Tumor DNA (ctDNA) and Epigenetic Biomarkers

Recent advances in medical research have brought about a new understanding through the study of ctDNA and epigenomic biomarkers. Circulating free DNA (cfDNA), which originates from healthy and cancerous tissues undergoing apoptosis or necrosis, has displayed significant potential in the field of oncology. In contrast, circulating tumor DNA (ctDNA), originating exclusively from tumor cells, carries somatic mutations and represents only a tiny portion of cfDNA [108].

This discovery offers a new prospect for previously untreated PM patients. In 2018, Hylebos and colleagues performed a comprehensive analysis of 10 PM patients using whole-exome sequencers (WES) to identify cancer-specific mutations, both in a germ line and in tumor DNA [109]. They were able to detect these mutations in serum samples from five treatment-naive patients using digital droplet PCR (ddPCR), and achieved a detection rate of sixty per cent. Interestingly, no tumor-specific alterations were observed in cfDNA from chemotherapy patients. Although ctDNA has the potential to be used as a biomarker of treatment response, further validation and cost-effective technologies will be required before it can be widely used in routine clinical practice.

Additionally, epigenetic modifications occurring during tumor development have emerged as promising biomarkers that are detectable in various body fluids. In asbestos-induced carcinogenesis, the generation of reactive oxygen species (ROS) leads to gene promoter methylation, orchestrated by poly(ADP-ribose) polymerase 1 (PARP1) and DNA (cytosine-5) methyltransferase 1 (DNMT1) [110]. Intriguingly, these epigenetic biomarkers have the potential to serve as novel therapeutic targets.

Nocchi et al. introduced an innovative approach by combining two epigenetically regulated markers, miR-126 and TM, with SMRP [111]. In fact, it has been reported that epigenetic mechanisms can silence TM gene expression in PM tissue, and hypermethylation of the miR-126 promoter region contributes to its downregulation. Despite a sensitivity rate of 60%, the authors reported that circulating methylated TM DNA effectively differentiated PM patients from the controls with a specificity of 82%. This finding complements the performance of miR-126 and SMRP as independent biomarkers for PM detection [112].

More recently, Guarrera et al. harnessed a genome-wide methylation array to identify distinct methylation patterns at selected CpGs in DNA extracted from white blood cells (WBC) in a cohort of 163 PM patients and 137 controls [113]. This discovery holds the promise of shedding further light on the epigenetic landscape of PM and its potential implications for diagnosis and treatment.

### 4.7. High-Mobility Group Box 1 (HMGB1)

HMGB1 is damage-associated molecular pattern protein that is released in the extracellular space during necrosis [37,73]. It has been considered to be a promising biomarker, with a cytoplasmic hyperacetylated isoform that can be released into the extracellular space, performing better than the unacetylated form present in the nucleus [25,36]. However, a subsequent investigation at the University of Liverpool revealed concerns regarding the integrity of the mass spectrometry data that was contributed by one of the authors of this study [114].

Few studies that show a high accuracy of the marker in terms of distinguishing asbestosis patients from healthy patients with and without asbestos exposure are present in the literature (AUC_ROC_: 0.88) [115]. However, no differences were found when patients with PM were compared with those with asbestosis (AUC_ROC_: 0.56), and no studies comparing this with other malignancies are available [25,92]. HMGB1 may be a potential target for PM, with some animal studies suggesting a possible role in this context (Table 3) [89].

### 4.8. Other Blood Biomarkers

Several other molecules have been evaluated as biomarkers of PM, with a few studies assessing their diagnostic performance [40,73].

Thioredoxin-1 (TRX-1) is a small protein involved in decreasing reactive oxygen species levels [116]. Its expression is increased in patients with PM, but its specificity is suboptimal (77.6%) [117].

Vascular endothelial growth factor (VEGF) is a marker of tumor angiogenesis; serum/plasma levels are increased in PM compared with patients with non-malignant asbestos-related disease [93,118,119]. Studies have demonstrated a possible role of VEGF as a prognostic and monitoring biomarker [39].

Integrin-linked kinase, protein ENOX2, hyaluronic acid, interleukin-6, circulating fibrinogen, chemokine RANTES, vimentin, matrix metalloproteinases, fibroblast growth factor, platelet-derived growth factor, hepatocyte growth factor, and tissue inhibitor of metalloproteinases represent other, less studied biomarkers [25,39,40,73,120].

### 4.9. Combination of Serum Biomarkers

The combination of multiple biomarkers might potentially increase accuracy in the screening, diagnosing, and monitoring of PM compared to single markers [40,113]. Combinations of several panels have been tested. In a prospective evaluation, the combination of blood calretinin and mesothelin yielded an AUC_ROC_ of 0.83 and a specificity of 98% [103].

Combining mesothelin and osteopontin did not increase the diagnostic accuracy, but a positive correlation with disease outcome was found when comparing patients with PM to patients with pleural plaques and to healthy individuals with and without asbestos exposure [96,121].

Adding carcinoembryonic antigen (CEA), chitinase-like protein, and cytokeratin 19 fragments improved the diagnostic accuracy of mesothelin [25,122,123]. Bonotti et al., who explored different combinations of biomarkers, found that the two best three-marker combinations were interleukin6–osteopontin–SMRP and IL6–osteopontin–desmin, with AUC_ROC_ of 0.945 and 0.95, respectively). The best four-marker combination was represented by SMRP–osteopontin–IL6–vimentin (AUC_ROC_ of 0.96) [124].

## 5. Conclusions and Future Perspectives

An accurate and early diagnosis of PM is increasingly desirable given the difficulty of treating advanced disease; the growing number of cases related to familial BAP1 germline mutations; and the discovery of other carcinogenic fibers, such as erionite [125].

**Figure 1:**
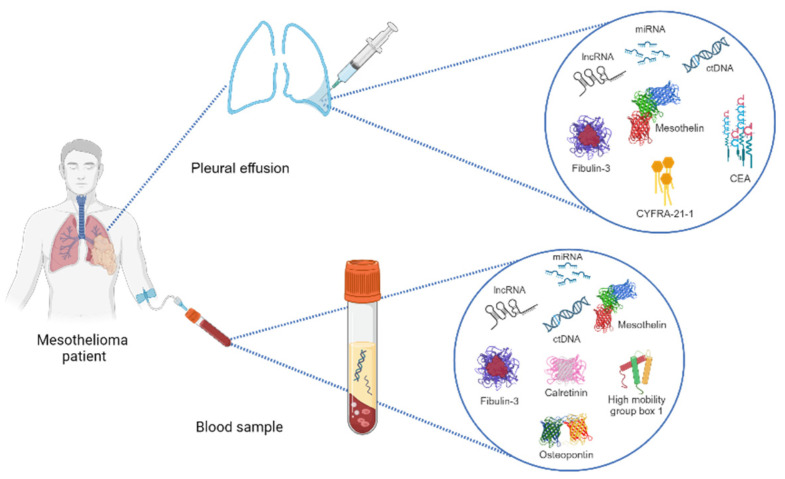
Representative scheme of diagnostic methods for analyzing biomarkers in pleural mesothelioma patients (see text and Table 2 for their diagnostic accuracy).

There are several biomarkers that can be identified in pleural fluid, tissue, and serum/plasma (Figure 1), all of which have the potential to enable the early diagnosis of PM, as well as to guide and evaluate the responses to therapy. None of the biomarkers explored herein, however, are ready for prime time, and future research will help us to clarify their roles in the care of PM patients. To date, mesothelin and SMRPs appear to be the most reliable markers, both in plasma and pleural fluid. As of October 2023, MESOMARK® (Fujirebio Diagnostics, Inc., Malvern, PA, USA) is the only FDA-approved biomarker test for mesothelioma. It is an enzyme-linked immunosorbent assay (ELISA) for the quantitative measurement of SMRPs in human serum.

Some biomarkers, such as serum HA, appear to be suboptimal due to rapid clearance from the systemic circulation, while it is unclear why fibulin-3 has higher diagnostic accuracy in plasma compared to pleural effusion. Osteopontin is a biomarker with validated prognostic power.

Some studies are currently ongoing on serum and/or pleural effusion biomarkers to detect PM early in patients exposed to asbestos or vermiculite (NCT02029105, NCT00897247).

Another interesting ongoing study (NCT04106973) aims to identify PM markers using a non-invasive technique that samples volatile organic compounds (VOCs) in the breath. The exhaled VOC profiles from subjects with histologically confirmed PM will be compared to matched control subjects with bilateral pleural plaques or bilateral pleural thickening.

There is also ongoing laboratory research that is examining biomarkers of angiogenesis and disease in patients with unresectable PM (NCT00898547).

Finally, a prospective study is collecting biopsied tissue to evaluate a new method for determining the stage and prognosis of individuals with PM (NCT03683680).

Internationally, large archives of high-quality specimens (i.e., biobanks) from patients with early-stage disease might significantly enhance the scientific research in this field [25,40,126].

Larger, prospective studies with appropriately standardized control groups could lead to more reliable data from single and multiple biomarkers. Indeed, the combination of biomarkers from different matrices might overcome the shortcomings related to matrix-specific markers [25,40].

Finally, the use of non-invasive markers from other matrices, e.g., breath analysis, represents the most promising perspective. Breathomics, i.e., the study of volatile organic compounds contained in the breath, is an increasingly investigated research area and might be used to screen patients at risk for PM, i.e., workers exposed to asbestos. Preliminary studies on the early stages of the disease have shown interesting findings, but the small sample size and the lack of an external validation mean that they are not yet generalizable [25,40].

## Figures and Tables

**Table 1:** Main methods used to identify biomarkers with relative advantages and disadvantages [23].

Method	Advantages	Disadvantages
Antigen-antibody reaction (eg ELISA)	Fast to perform Inexpensive reagents and equipment Easy to perform with simple procedure Effective for soluble substances such as proteins, hormones, peptides, and antibodies	Risk of cross-reactivity High possibility of false positive/negative Wells to be read quickly due to short-term enzyme/substrate reaction Inadequate for microRNAs
Proteomics	Comprehensive analysis of proteins Identifies protein modifications Can uncover novel biomarkers Provides insights into functional changes	Technical expertise needed Expensive equipment Data analysis complexity Limited to known proteins
Genomics	Provides genetic information Identifies gene mutations Potential for early detection Can be used for risk assessment	Limited to DNA mutations May not capture all changes Complex data analysis Incomplete understanding
Transcriptomics	Captures gene expression levels Reveals potential therapeutic targets Helps to identify novel biomarkers Can distinguish subtypes	RNA instability Data analysis challenges Limited to RNA-based markers May not reflect true protein levels
Metabolomics	Provides insight into metabolic changes Identifies unique metabolic profiles Potential for early detection Can offer insights into treatment response	Metabolite variation Data interpretation Limited coverage of metabolites Technical challenges
Immunohistochemistry (IHC)	Validates biomarkers in tissue Useful for clinical applications Identifies protein expression Well-established technique	Limited to known targets Semi-quantitative information Inter-observer variability Dependent on tissue availability and quality
MicroRNA Profiling	Captures microRNA expression Useful for diagnostic and prognostic purposes Potential for early detection Minimally invasive samples are enough	Specific to microRNAs Data analysis complexity May require validation studies Limited to known microRNAs
Next-Generation Sequencing (NGS)	Comprehensive analysis of various data types Can integrate genomics, transcriptomics, and more Provides holistic view of molecular changes Identifies novel biomarkers	Data volume and complexity Requires cost and technical expertise Presents bioinformatics challenges Demands large sample sizes

**Table 2:** Summary of the main pleural and blood biomarkers of PM, related to their reported accuracy values. When available, pooled data of accuracy from meta-analyses are reported. AUC_ROC_: area under the ROC curve; PE: pleural effusion; AE: asbestos-exposed; MPF: megakaryocyte potentiating factor; HMGB1: high-mobility group box 1.

Matrix	Biomarker	Cut-Off	AUC_ROC_	Sensitivity	Specificity
Pleural	Mesothelin	1.96–2.5 nM	0.83 [25,26,27]	44–100%	46–100%
	Fibulin-3	346.01–378.33 ng/mL	0.68–0.97 [25,28]	78.4–83.8%	92.4–97.6%
	Hyaluronic acid	100,000 ng/mL	0.78–0.83 [25,29]	44.0%	96.5%
	Cell-free microRNAs	-	0.92 (miR-143 + miR-210 + miR-200c) [30]	92.3%	96.1%
	CYFRA-21-1 vs. benign PE	71.5 ng/mL	0.76 [31]	76%	85%
	CYFRA-21-1 vs. other cancers	100 ng/mL	0.61 [31]	81%	47%
	CEA vs. benign PE	5.5 ng/mL	0.32 [31]	30%	98%
	CEA vs. other cancers	3.8 ng/mL	0.20 [31]	98%	56%
Blood	Mesothelin vs. healthy	-	0.87 [27]	66%	97%
	Mesothelin vs. other cancers	-	0.73 [27]	60%	81%
	Mesothelin vs. benign AE	-	0.84 [27]	58%	89%
	Calretinin	0.42 ng/mL	0.86 [32]	71%	95%
	Calretinin + Mesothelin	0.60–0.85 ng/mL; 2.32–2.91 nM	0.83 [33]	46–66%	98–99%
	MPF	12.38 ng/ml	0.78 [25,34,35]	68%	95%
	Osteopontin vs. healthy	48.3 ng/mL	0.89 [24,36,37]	65%	81%
	Fibulin-3	52 ng/mL	0.91 [25]	62%	82%
	miR-197-3p	-	0.76 [38,39,40]	-	-
	miR-625-3p		0.80 [41]	73.3%	78.6%
	miR-20a		0.98 [38,39,40]	-	-
	miR-126It		0.74 [42]	71%	69%
	miR-132-3p + miR-126	-	0.76 [41]	77%	86%
	miR-103a-3p + miR-30e-3p	-	0.94 [43]	95.5%	80%
	ATG5	23 ng/mL	0.81 [44]	43%	98%
	GAS5	-	0.86 [45]	64–73%	97%
	ARSA mRNA.1	-	0.94 [46]	90%	93.7%
	DRAM mRNA	-	0.82 [46]	78.3%	87.5%
	hsa-miR-2053	-	0.91 [46]	85%	97.5%
	LncRNA-RP1-86D1.3	-	0.88 [46]	83.3%	95%
	HMGB1 vs. healthy	52.2 ng/mL	0.88 [25]	100%	88.3%
	HMGB1 vs. asbestosis	52.3 ng/mL	0.56 [25]	100%	29.3%

**Table 3:** Summary of potential treatment options based on biomarkers related to the study type. CAR-T: chimeric antigen receptor-T; HMBG1: high-mobility group box 1.

Targeted Biomarker	Tested Treatment Options	Study Type
Mesothelin [73]	Chimeric monoclonal antibodies (Amatuximab)	Human, phase II
	Antibody–drug conjugates Anetumab ravtansine (BAY94-9343) BMS-986148 BAY2287411	Human, phase I, II phase II phase I
	Immunotoxins SS1P LMB-100	Human, phase I phase I, II
	Cancer vaccines CRS-207	Human, phase I, II
	Mesothelin-targeted cellular therapy CAR-T	Human phase I, II
Fibulin-3 [88]	Anti-Fibulin-3 monoclonal antibody	Preclinical (animal)
HMBG1 [89]	Polypeptides Recombinant HMG Box-A Anti-HMGB1 neutralizing monoclonal antibody Chemical pharmaceuticals Ethyl pyruvate Aspirin and its metabolite, salicylic acid Plant extract Flaxseed lignans	Preclinical (animal)
microRNA [90,91,92,93]	TargomiRs (miR-16 mimic-drug delivery vehicle) miR-206 miR-215-5p miR-486-5p miR-16, mi-16-5p miR-126 miR-193a-3p	Human, phase 1 Preclinical (cell lines, animal) Preclinical (cell lines, animal) Preclinical (cell lines) Preclinical (animal)

## Data Availability

No new data were created or analyzed in this study. Data sharing is not applicable to this article.
